# Supplementary material for: Plasma Amino Acids and Acylcarnitines Are Associated with the Female but Not Male Adolescent Swimmer’s Performance: An Integration between Mass Spectrometry and Complex Network Approaches
Source: Biology (Basel). 2022 Nov 29;11(12):1734. doi: 10.3390/biology11121734 (PMC9774704; doi:10.3390/biology11121734)
Supplement: Supplementary file 1 [file biology-11-01734-s001.zip › Figure S1.pdf]

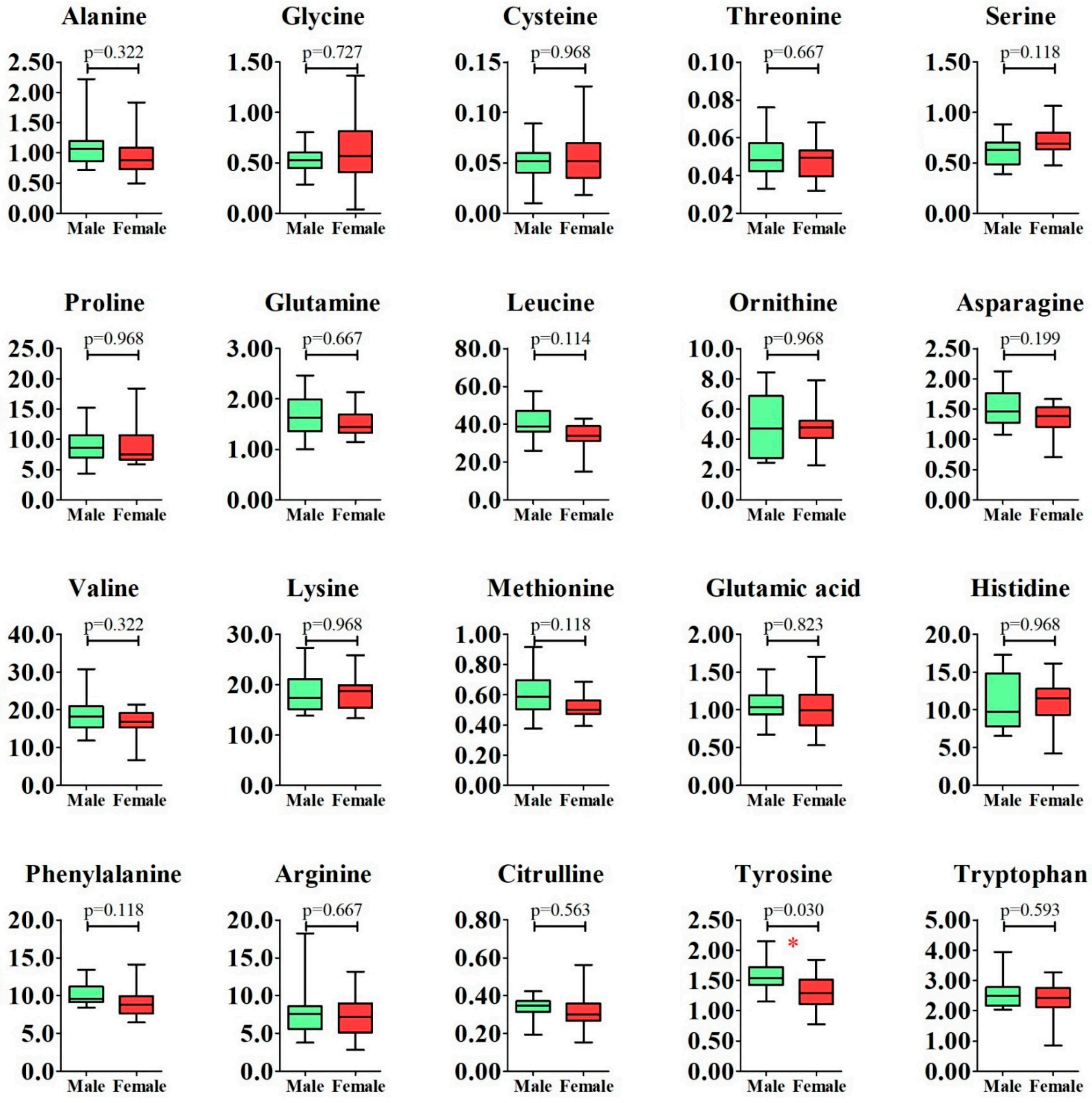

Supplementary Figure S1. Comparison of plasma amino acids between male and female adolescent swimmers by the false discovery rate. Values are expressed as normalized intensity.
