# Supplementary material for: Plasma Amino Acids and Acylcarnitines Are Associated with the Female but Not Male Adolescent Swimmer’s Performance: An Integration between Mass Spectrometry and Complex Network Approaches
Source: Biology (Basel). 2022 Nov 29;11(12):1734. doi: 10.3390/biology11121734 (PMC9774704; doi:10.3390/biology11121734)
Supplement: Supplementary file 1 [file biology-11-01734-s001.zip › Table S1.pdf]

**Supplementary Table S1.** Optimization of multiple reaction monitoring transitions were for each compound analyzed.

| Compound name | Parent (*m/z*) | Daughter (*m/z*) | Dwell (s) | Cone (V) | Collision (V) |
|---|---|---|---|---|---|
| Glycine | 76.0000 | 30.1000 | 0.030 | 20 | 7 |
| Alanine | 90.1000 | 44.1000 | 0.030 | 20 | 8 |
| Serine | 106.1000 | 60.0000 | 0.030 | 14 | 8 |
| Proline | 116.1000 | 70.0000 | 0.030 | 20 | 10 |
| Valine | 118.1000 | 72.1000 | 0.030 | 20 | 10 |
| Threonine | 120.1000 | 74.0000 | 0.030 | 38 | 20 |
| Cysteine | 122.1000 | 105.0000 | 0.030 | 20 | 10 |
| Glutamine | 130.0000 | 84.1000 | 0.030 | 20 | 8 |
| Leucine | 132.1000 | 85.1000 | 0.030 | 20 | 9 |
| Ornitine | 133.0000 | 70.0000 | 0.030 | 15 | 14 |
| Asparagine | 133.1000 | 87.0000 | 0.030 | 20 | 6 |
| Aspartic Acid | 134.1000 | 88.0000 | 0.010 | 14 | 10 |
| Lysine | 147.1000 | 84.0000 | 0.030 | 14 | 14 |
| GlutamicAcid | 148.1000 | 130.0000 | 0.030 | 20 | 8 |
| Methionine | 150.0000 | 104.0000 | 0.030 | 20 | 9 |
| Histidine | 156.1000 | 110.0000 | 0.030 | 20 | 16 |
| Freecarnitine | 162.0000 | 85.0000 | 0.030 | 40 | 19 |
| C0 | 162.1000 | 85.0000 | 0.030 | 40 | 19 |
| HidroFC | 165.1000 | 85.0000 | 0.030 | 40 | 19 |
| Phenylalanine | 166.1000 | 120.1000 | 0.030 | 20 | 12 |
| Arginine | 175.1000 | 70.1000 | 0.030 | 30 | 21 |
| Citrulline | 176.0000 | 113.0000 | 0.030 | 20 | 14 |
| Tyrosine | 182.1000 | 136.1000 | 0.030 | 20 | 12 |
| C2 | 204.1000 | 85.0000 | 0.030 | 35 | 19 |
| Tryptophan | 205.1000 | 146.0000 | 0.030 | 16 | 18 |
| C3 | 218.1000 | 85.0000 | 0.030 | 35 | 19 |
| C4 | 232.1000 | 85.0000 | 0.030 | 35 | 19 |
| C5 | 246.1000 | 85.0000 | 0.030 | 35 | 19 |
| C6 | 260.0000 | 85.0000 | 0.030 | 35 | 19 |
| C8 | 288.1000 | 85.0000 | 0.030 | 40 | 19 |
| C10 | 316.0000 | 85.0000 | 0.030 | 40 | 19 |
| C12 | 344.0000 | 85.0000 | 0.030 | 40 | 19 |
| C14 | 372.0000 | 85.0000 | 0.030 | 40 | 28 |
| C16 | 400.3000 | 85.0000 | 0.030 | 40 | 25 |
| C16OH | 416.0000 | 85.0000 | 0.030 | 40 | 25 |
| C18:1 | 426.0000 | 85.0000 | 0.030 | 40 | 19 |
| C18 | 428.0000 | 85.0000 | 0.030 | 40 | 19 |
