# Supplementary material for: Plasma Amino Acids and Acylcarnitines Are Associated with the Female but Not Male Adolescent Swimmer’s Performance: An Integration between Mass Spectrometry and Complex Network Approaches
Source: Biology (Basel). 2022 Nov 29;11(12):1734. doi: 10.3390/biology11121734 (PMC9774704; doi:10.3390/biology11121734)
Supplement: Supplementary file 1 [file biology-11-01734-s001.zip › Table S2.pdf]

Supplementary Table S2. Correlations among amino acids and the swimmers' performances over distances and the critical velocity (CV).

	Male					Female				
	**100-m**	**200-m**	**400-m**	**800-m**	**CV**	**100-m**	**200-m**	**400-m**	**800-m**	**CV**
**Alanine**	-0.38	-0.39	-0.28	-0.33	0.32	-0.01	0.13	0.17	0.09	-0.05
**Glycine**	-0.07	0.04	-0.05	-0.05	-0.00	0.16	0.13	0.17	0.13	-0.15
**Cysteine**	0.06	0.17	0.08	0.02	-0.02	0.14	0.08	0.03	0.02	-0.05
**Threonine**	0.02	0.07	-0.04	-0.06	0.04	-0.03	0.22	0.06	0.11	-0.10
**Serine**	-0.05	-0.02	-0.24	-0.19	0.23	-0.06	-0.16	-0.26	-0.17	0.18
**Proline**	-0.03	0.04	-0.15	-0.11	0.06	-0.02	0.03	0.03	0.01	0.04
**Glutamine**	0.14	-0.07	0.02	0.07	-0.11	0.10	0.06	0.12	0.07	-0.09
**Leucine**	-0.13	-0.07	-0.17	-0.17	0.16	-0.19	-0.08	-0.16	-0.06	0.08
**Ornithine**	0.04	0.18	-0.02	-0.05	0.03	0.18	0.09	0.03	0.11	-0.10
**Asparagine**	-0.17	-0.11	-0.19	-0.20	0.18	-0.23	-0.16	-0.25	-0.15	0.17
**Valine**	-0.03	-0.00	-0.01	-0.01	-0.01	-0.08	-0.03	-0.14	-0.06	0.10
**Lysine**	0.07	0.11	0.08	0.04	-0.08	0.10	-0.04	0.07	0.08	-0.11
**Methionine**	-0.10	-0.12	-0.05	-0.07	0.03	0.09	0.23	0.19	0.18	-0.17
**Glutamic acid**	0.11	0.06	0.17	0.09	-0.13	0.13	0.09	0.14	0.09	-0.11
**Histidine**	0.26	0.31	0.22	0.20	-0.22	0.03	-0.03	-0.03	0.01	-0.01
**Phenylalanine**	-0.13	-0.27	-0.05	-0.12	0.11	0.17	0.30	0.28	0.36	-0.38
**Arginine**	0.29	0.24	0.26	0.27	-0.29	0.17	0.30	0.28	0.36	-0.38
**Citrulline**	0.26	0.24	0.12	0.24	-0.30	0.08	-0.16	-0.10	-0.03	0.07
**Tryptophan**	0.28	0.32	0.34	0.21	-0.19	0.10	0.06	0.12	0.07	-0.09
**Tyrosine**	0.08	-0.02	0.09	0.06	-0.04	0.48*	0.41	0.47*	0.52*	-0.51*
