# Supplementary material for: Plasma Amino Acids and Acylcarnitines Are Associated with the Female but Not Male Adolescent Swimmer’s Performance: An Integration between Mass Spectrometry and Complex Network Approaches
Source: Biology (Basel). 2022 Nov 29;11(12):1734. doi: 10.3390/biology11121734 (PMC9774704; doi:10.3390/biology11121734)
Supplement: Supplementary file 1 [file biology-11-01734-s001.zip › Table S3.pdf]

Supplementary Table S3. Correlations among acylcarnitines and the swimmers' performances over distances and the critical velocity (CV).

	Male					Female				
	**100-m**	**200-m**	**400-m**	**800-m**	**CV**	**100-m**	**200-m**	**400-m**	**800-m**	**CV**
**Free carnitine**	0.22	0.34	0.22	0.14	-0.13	0.10	0.06	0.12	0.08	-0.09
**C2**	0.13	0.28	0.18	0.23	-0.28	0.13	0.06	0.09	0.09	-0.11
**C3**	0.08	0.03	0.22	0.26	-0.27	0.03	-0.18	-0.04	0.03	-0.04
**C4**	0.17	-0.09	0.01	0.13	-0.12	0.35	0.09	0.26	0.25	-0.27
**C5**	-0.31	-0.22	-0.24	-0.27	0.27	0.06	0.08	0.01	0.13	-0.11
**C6**	0.28	0.32	0.16	0.23	-0.21	0.14	0.24	0.06	0.16	-0.17
**C8**	0.16	0.24	0.01	0.08	-0.06	0.17	0.20	0.19	0.16	-0.19
**C10**	0.15	0.29	0.01	0.07	-0.05	-0.03	-0.14	-0.12	-0.04	0.04
**C12**	0.07	0.21	-0.07	-0.01	0.00	-0.18	-0.43	-0.31	-0.28	0.27
**C14**	-0.13	0.03	-0.19	-0.11	0.12	-0.19	-0.42	-0.33	-0.29	0.29
**C16**	-0.16	-0.00	-0.22	-0.18	0.19	-0.21	-0.46	-0.31	-0.26	0.24
**C18**	-0.16	0.01	-0.32	-0.23	0.21	-0.23	-0.27	-0.26	-0.19	0.16
